# Advancements, Challenges, and Future Directions in Tackling Glioblastoma Resistance to Small Kinase Inhibitors

**DOI:** 10.3390/cancers14030600

**Published:** 2022-01-25

**Authors:** Federica Fabro, Martine L. M. Lamfers, Sieger Leenstra

**Affiliations:** Department of Neurosurgery, Brain Tumor Center, Erasmus University Medical Center, 3015 CN Rotterdam, The Netherlands; f.fabro@erasmusmc.nl (F.F.); m.lamfers@erasmusmc.nl (M.L.M.L.)

**Keywords:** drug resistance, small kinase inhibitors, glioblastoma, cell culture models, overcoming resistance

## Abstract

**Simple Summary:**

Drug resistance is a major issue in brain tumor therapy. Despite novel promising therapeutic approaches, glioblastoma (GBM) remains refractory in showing beneficial responses to anticancer agents, as demonstrated by the failure in clinical trials of small kinase inhibitors. One of the reasons may lie in the development of different types of drug resistance mechanisms derived from the intrinsic heterogeneous nature of GBM. Obtaining insights into these mechanisms could improve the management of the clinical intervention and monitoring. Such insights could be achieved with the improvement of preclinical in vitro models for studying drug resistance.

**Abstract:**

Despite clinical intervention, glioblastoma (GBM) remains the deadliest brain tumor in adults. Its incurability is partly related to the establishment of drug resistance, both to standard and novel treatments. In fact, even though small kinase inhibitors have changed the standard clinical practice for several solid cancers, in GBM, they did not fulfill this promise. Drug resistance is thought to arise from the heterogeneity of GBM, which leads the development of several different mechanisms. A better understanding of the evolution and characteristics of drug resistance is of utmost importance to improve the current clinical practice. Therefore, the development of clinically relevant preclinical in vitro models which allow careful dissection of these processes is crucial to gain insights that can be translated to improved therapeutic approaches. In this review, we first discuss the heterogeneity of GBM, which is reflected in the development of several resistance mechanisms. In particular, we address the potential role of drug resistance mechanisms in the failure of small kinase inhibitors in clinical trials. Finally, we discuss strategies to overcome therapy resistance, particularly focusing on the importance of developing in vitro models, and the possible approaches that could be applied to the clinic to manage drug resistance.

## 1. Introduction

Glioblastoma (GBM) is a deadly brain cancer, classified by WHO as a grade IV astrocytoma, which remains one of the most challenging cancers to treat [1]. In fact, despite clinical intervention consisting of surgery and chemoradiation, the median overall survival ranges between 14.6 and 16.7 months [2,3]. Only recently, the addition of tumor-treating fields (TTF) has been associated with improvement in the median survival up to 20.9 months [4]. The reason of this fatal outcome resides for a great part in therapy resistance. Cancer chemotherapy resistance comprises several aspects of the innate and acquired ability of the tumor to evade the effects of therapy and it is described as a multifactorial phenomenon.

Up to now, GBM has displayed the ability to resist both conventional and novel targeted treatments. This is also the case for small kinase inhibitors which have shown great advances in many other types of cancer. These compounds act on protein kinases which promote cell proliferation, survival, and migration, constituting key players in tumor development and potential targets for anticancer therapy [5,6,7]. Despite being beneficial for various malignancies such as chronic myeloid leukemia (CML), non-small-cell lung cancer (NSCLC), renal cell carcinoma (RCC), breast cancer, hepatocellular carcinoma, colorectal cancer, gastrointestinal stromal tumors (GIST), thyroid cancer, and melanoma, none of these compounds has been approved for GBM treatment [8,9,10].

The development of strategies that tackle resistance is of utmost importance to improve the efficacy of the therapies. Due to the large molecular heterogeneity among tumors and the complexity of tumor progression, identifying the best strategy to overcome drug resistance is particularly challenging [11]. Therefore, understanding the underlying cause of resistance is the initial step to overcome this challenge. The development of in vitro models that accurately recapitulate the GBM biology is the first important step to better predict clinical behavior and drug response, including resistance [12].

With this review, we aimed to (I) explore the heterogenous resistance mechanisms in GBM, primarily for small kinase inhibitors, which are the most exploited targeted therapy in cancer; (II) address the relevance of designing preclinical in vitro drug resistance models; (III) discuss the possible clinical strategies in overcoming the problem of drug resistance.

## 2. Heterogeneity of GBM

Glioblastoma was formerly known as glioblastoma multiforme. As the word “multiforme” implies, glioblastoma has multiple forms, and its diversity is evident at various levels. Macroscopically, it presents with areas of necrosis, abnormal vascularization, and thrombi. Histologically, it is possible to distinguish presence of microvascular proliferation and necrotic foci surrounded by pseudopalisading cells. Moreover, tumor cells show great pleomorphism, some containing intranuclear inclusions and others resembling adipocytes due to the presence of lipomatous vacuoles [13,14]. At the molecular level, the range of genetic alterations is wide. Three molecular defined subtypes based on gene expression profiles have been described: classical, mesenchymal, and proneural [15,16]. Classifying GBM into subgroups has been an attempt to first describe its heterogeneous nature. However, the heterogeneity that underlies GBM is deeper and more complex. Subsequent studies demonstrated the presence of multiple subtypes within the same tumor, introducing the concept of intratumoral heterogeneity. As a matter of fact, Sottoriva et al. observed not only different subpopulations within the same tumor, but also their divergent evolution in time, which is likely to be at the root of therapy failure [17]. With the advent of the single cell sequencing technology, the intratumoral heterogeneity has reached another level of investigation. The GBM subtype classifiers are variably expressed across individual cells within a tumor [18]. In addition, four dynamic and interchangeable cellular states (OPC-like, NPC-like, AC-like, and MES-like) have been identified, which recapitulate distinct neural progenitor cell types and are linked to specific genetic drivers [19].

Studies on paired primary and recurrent gliomas reveal that intratumoral heterogeneity also drives the genomic evolution of the tumor toward recurrence after treatment [20,21,22]. Of relevance, the genomic profiles of GBMs recurring at a distance of the primary tumor shared only a minority (33%) of the initial tumor mutations, in contrast with locally recurring tumors which shared a majority (57%) of the initial mutations [21].

Another source of complexity influencing heterogeneity is given by the tumor microenvironment (TME) [23]. TME is not constituted only by cancerous cells, but also by different populations of immune cells, stromal cells, endothelial cells, and pericytes, creating different types of niches within the tumor [24]. There is growing evidence that in these niches, different tumor cell types (proliferating, infiltrating, cancer stem cell (CSC)-like) and different noncancerous cells (astrocytes, microglia, macrophages, dendritic cells, lymphocytes) dynamically reshape different parts of the tumor [25]. In particular, astrocytes represent the most abundant glial cell population in contact with GBM. These cells display a reactive phenotype when in contact with tumor cells, expressing a high level of GFAP [26]. This population has been demonstrated to augment GBM malignancy by causing aberrant cell proliferation and enhancing migration [26]. In addition, this interaction could also promote a release of anti-inflammatory cytokines such as TGF-β, IL-10, and G-CSF, contributing to anti-inflammatory responses and creation of an immunosuppressive environment [27]. Brain tumor endothelial cells (ECs) have a major role in tumor growth and invasion as evidence reports the functional crosstalk between the tumor and ECs [28,29]. GBM orchestrate vascular niches that maintain the cancer stem cells (CSC) pool which, in turn, produces VEGF to promote tumor angiogenesis [30,31]. Tumor microenvironment is also characterized by the presence of immune cells. Particularly, tumor-associated macrophages (TAMs) are the dominant infiltrating immune cell population [32]. TAMs display a heterogeneous spectrum of phenotypes, of which extremes exert either antitumor (M1-like) or protumor functions (M2-like) [33].

Heterogeneity is one of the main issues that make GBM treatment challenging and it is also the key to understand the complexity of therapy resistance to standard and experimental therapies.

## 3. Temozolomide Resistance

The standard-of-care therapy for GBM includes surgery followed by radiotherapy as well as concomitant and maintenance temozolomide (TMZ) chemotherapy [34]. TMZ is a small (194 Da) and lipophilic antineoplastic agent that alkylates DNA. Its stability at low pH and lipophilicity allow it to have high oral bioavailability and the important ability to cross the blood–brain barrier [35,36]. At the molecular level, its cytotoxic effect is mediated by the addition of methyl groups on the DNA. Specifically, the most common lesions are the formation of N7-methylguanine (7meG, 70%), 3-methyladenine (3meA, 10%), and O6-methylguanine (O6meG, 7%) [37]. Even though the O6meG mutation is accounted only for a small proportion, it is considered to be the most cytotoxic and mutagenic lesion caused by TMZ [38]. During DNA replication, mispairs arise, leading to the activation of the mismatch repair (MMR) system. However, the MMR complex is not capable of resolving the lesion correctly, leading to a futile MMR cycle [39]. This generates additional mutations and DNA damage, such as double strand breaks (DSBs), causing the ultimate cytotoxic and apoptotic effects [40,41]. 

**Figure 1:**
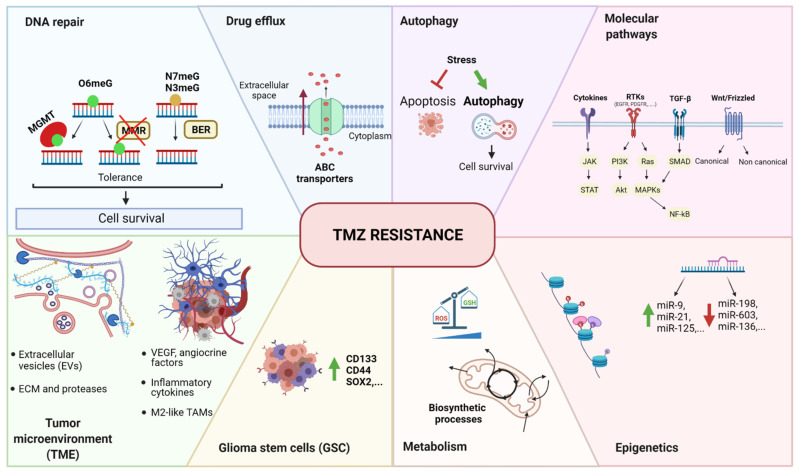
Summary of temozolomide resistance mechanisms in GBM. The main mechanisms can be grouped as follows: DNA repair, drug efflux, autophagy, molecular pathways, epigenetics, metabolism, glioma stem cells (GSC), and tumor microenvironment (TME). O6meG: O6-methylguanine, N7meG: N7-methylguanine, N3meG: N3-methylguanine, MGMT: O6-methylguanine-DNA methyltransferase, MMR: mismatch repair, BER: base excision repair, ABC transporter: ATP-binding cassette transporter, RTK: tyrosine kinase receptor, TGF-β: tumor growth factor β, Wnt: wingless-related integration site, JAK: Janus kinase, STAT: signal transducer and activator of transcription; PI3K: phosphoinositide-3-kinase, Akt: protein kinase B (PKB), Ras: rat sarcoma virus, MAPK: mitogen-activated protein kinases, NF-κB: nuclear factor kappa-light-chain-enhancer of activated B cells, miR: microRNA, ROS: reactive oxygen species, GSH: glutathione, CD133: cluster of differentiation 133, CD44: cluster of differentiation 44, SOX2: (sex-determining region Y)-box 2, VEGF: vascular endothelial growth factor, TAM: tumor-associated macrophages, ECM: extracellular matrix, TMZ: temozolomide.

As it is an alkylating agent, the primary damage of TMZ occurs at the DNA level, but the subsequent effects reverberate on a wide range of cellular events. The same concept can also be applied to resistance mechanisms. GBM, in fact, can escape TMZ toxicity through a broad range of different processes (Figure 1). The most direct mechanism of resistance is the activation of DNA repair [42]. In particular, O6-methylguanine-DNA methyltransferase (MGMT) is an essential enzyme that removes O6meG adducts induced by TMZ and neutralizes its cytotoxic effects [43,44]. Furthermore, GBM is intrinsically equipped with the plasticity necessary to develop and acquire enhanced survival features [45]. At the intracellular level, the mechanisms by which GBM resists TMZ include drug efflux through ABC transporters, blockade of apoptosis and concomitant activation of autophagy, intracellular signaling cascade adaptation, epigenetic modulation (including microRNA, histone modifications, and methylation), and metabolic reprogramming through the reduction of reactive oxygen species (ROS) and the activation of biosynthetic processes. The presence of glioma stem cells (GSCs), which are considered intrinsically resistant to therapies, also influences the tumor response to the therapy. At the TME level, cellular components such as astrocytes, endothelial cells, and immune cells modulate the surrounding environment through the production of pro-survival signals. In parallel, noncellular factors like extracellular matrix (ECM) rearrangements, release of extracellular vesicles (EVs), and hypoxia contribute to the protumoral environment and support adaptation to the drug. We have summarized these main findings in Figure 1, which are extensively discussed in other recent reviews [46,47].

The identification of this large variety of mechanisms points out the important role of heterogeneity as a driving factor in the generation of distinct resistance profiles. To make the situation more complex, resistance heterogeneity has been found to also take place within tumor subpopulations, potentially signifying the need for combinatorial therapy approaches to target different tumor cell populations [48,49].

## 4. Kinases in GBM

Kinases are crucial regulatory enzymes that maintain the signal transduction homeostasis in normal cells. However, when disrupted, they give rise to several diseases, including cancer [50]. Cellular functions such as proliferation, survival, apoptosis, motility, angiogenesis, metabolism, and evasion of immune response are all regulated by kinases and are frequently disrupted in tumors, including GBM [51,52]. GBM harbors a broad and diverse genomic landscape of mutations that often target kinases, leading to a variety of critical signaling disruptions [53]. For this reason, several small kinase inhibitors were tested as potential novel therapies [54]. The most disrupted kinases in GBM include receptor tyrosine kinases (RTK) such as epidermal growth factor (EGFR), platelet-derived growth factor (PDGFR), hepatocyte growth factor (MET), fibroblast growth factor (FGFR), vascular endothelial growth factor (VEGFR), and insulin-like growth factor 1 receptor (IGF1R). A multitude of downstream signaling cascades have been reported to be activated by these different RTKs. We will discuss the most common and dysregulated kinases involved in the downstream intracellular signaling pathways in GBM which comprise the RAS–RAF–MEK–ERK, PI3K–AKT–mTOR, PKC, JAK/STAT3, p53, and Rb pathways.

### 4.1. RTKs

#### 4.1.1. EGFR

The analysis of The Cancer Genome Atlas (TCGA) data demonstrated that genetic alterations in the epidermal growth factor receptor (EGFR) are the most frequent receptor tyrosine kinase (RTK) lesions in primary glioblastoma, occurring overall in 57% of these tumors [53]. Amplification of EGFR and an active mutant EGFRvIII also occur frequently in GBM and are mainly associated with the classical subtype [16]. Increased expression or alterations of EGFR have been associated with autocrine and paracrine loops which constitutively stimulate survival and proliferation signals [55,56,57]. EGFR mediates different pathways based on its localization. When EGFR is bound to the membrane, it propagates its signal through traditional pathways such as RAS/MAPK/ERK, PI3K/AKT, JAK/STAT, and phospholipase C (PLC)/PKC [58]. However, EGFR can also localize in the nucleus where it acts as a cofactor for transcription and in the mitochondria, leading to respiratory inhibition [59,60,61]. The mutant variant EGFRvIII shares the common signaling pathways with EGFR, but it also has a lower-level constitutive kinase activity and phosphorylates other kinases and receptors [62].

#### 4.1.2. PDGFR

Platelet-derived growth factor receptor (PDGFR) is the second most altered RTK in GBM, comprising 10–13% of tumors [53,63]. Amplification is the most common alteration, but other alterations like deletions, point mutations, and rearrangements are also found [64,65]. Similar to EGFR, PDGFR has a mutated ectopic form that is activated in the absence of the ligand. This mutant is termed PDGFR-α (Δ8,9) which presents a deletion of exons 8 and 9, coding for a portion of the extracellular domain [66]. Of note, another ligand-independent conformation derives from the fusion between PDGFRA and VEGFR2/KDR named KP fusion, predominantly localized in the cytoplasm and harboring elevated tyrosine kinase activity [64].

#### 4.1.3. MET

The hepatocyte growth factor receptor MET is highly expressed in GBM. Its genetic alterations have been found in 1.6–4% of the tumors [53,63]. Similarly to other RTKs, this receptor is involved in autocrine and paracrine loops which contribute to the sustainment of the cancer cells [67]. Through its signaling cascades, MET influences different and important cell functions: cell proliferation, invasion, cell survival, and angiogenesis [67,68]. A long list of different abnormalities has been found in GBM which include amplifications, overexpression, fusion genes, and specific mutations [69]. Navis et al. described an autoactivated variant characterized by the deletion of exons 7 and 8 (METΔ7−8), which encode for a portion of the Ig-like domain [70]. Fusion genes have been found only in secondary or pediatric GBMs [71,72].

#### 4.1.4. FGFR

According to the TCGA data, the fibroblast growth factor receptor (FGFR) is found altered in 3.2% of GBMs with a great variability in FGFR family expression [53,73]. Its signal cascade regulates important cell functions such as proliferation, survival, and cytoskeletal reorganization. Apart from FGF, its activity can be modulated by other cell surface proteins such as G-protein-coupled receptors (GPCRs), cell adhesion molecules (CAMs), and other RTKs [73]. Oncogenic chromosomal translocations that fuse the in-frame tyrosine kinase domains of the FGFR family genes to the transforming acidic coiled-coil (TACC) domains were reported as clonal events [74]. The FGFR–TACC fusion protein displays constitutive kinase activity with growth-promoting effects and induces mitotic and chromosomal segregation defects, causing more genomic instability [74,75].

#### 4.1.5. VEGF

Vascular endothelial growth factor (VEGF) is one of the most important angiogenic factors, and through the signaling mediated by its receptor VEGFR, it stimulates angiogenesis in tumors [76]. GBM is highly vascularized and expresses VEGFR1/FLT1 and VEGFR2/KDR on the cell surface. VEGFR2 is preferentially expressed on the cell surface of glioma stem-like cells (GSCs), conferring resistance to therapy and inducing proliferation of the tumor [77,78,79].

#### 4.1.6. IGF-1R

Overexpression of insulin-like growth factor 1 receptor (IGF1R) in GBM has been shown to be a poor prognostic factor. Its activity protects tumor cells against apoptosis and promotes their survival [80,81]. Thus far, inhibitors targeting IGF1R have advanced only to preclinical stage.

### 4.2. Downstream Intracellular Signaling

#### 4.2.1. RAS–RAF–MEK–ERK Pathway

The cascade events of this pathway result in the modulation of fundamental cellular functions such as proliferation, cellular growth, motility, and apoptosis. In high-grade gliomas, its upregulation takes part in the amplification of mitogenic stimuli [82,83]. In GBM, the signal is hyperactivated due to overexpression or increased activity of its upstream regulators, but rarely due to somatic mutations of the RAS and BRAF genes [84,85,86]. In line with these observations, the TCGA data reported 1% and 2% mutation rates of RAS and BRAF, respectively [53]. The most common active mutation is harbored by BRAF, named BRAF-V600E, which results in an activated protein that signals to MEK–ERK constitutively, thereby stimulating cell proliferation and survival [87]. The downstream activation of ERK is associated with poor outcomes and hence targeting the RAS–RAF–MEK–ERK pathway has been seen as a potential therapy for GBM patients [88].

#### 4.2.2. PI3K–AKT–mTOR Pathway

The PI3K network is a complex cascade of signals regulating different cellular processes, including proliferation, motility, differentiation, metabolism, and survival [89]. Around 90% of GBM are characterized by activation of the PI3K pathway which, interestingly, seems to have distinct roles in genetically identical cell populations but with different states of differentiation [90]. PI3K activity is blocked by PTEN, the loss of which is a hallmark of GBM and a very frequent event, with deletions occurring in over 90% of primary glioblastomas [16]. The activation of PI3K signaling is mostly triggered by PTEN loss or inactivation, but also activating mutations in PI3K have been found [91,92,93]. Mutations in AKT and mTOR are rare in GBM. Nevertheless, their increased activation through upstream activators make them a very attractive target for therapeutic intervention [94].

#### 4.2.3. PKC Pathway

Activation of protein kinase C (PKC) is one of the earliest events in a cascade that controls a variety of cellular responses depending on the isoform that is activated. PKCα exerts a pro-mitotic and prosurvival effect, while PKCβ is involved in glioma angiogenesis, proliferation, and resistance to apoptosis [95,96]. The role of the isoform PKCδ in gliomas depends on the phosphorylation site, leading to the activation of invasion or apoptosis processes [97]. Overexpression of PKCε in GBM is a marker of poor prognosis and it participates in cell-to-cell adhesion processes [97]. PKCη also contributes to increased GBM cells proliferation while PKCι promotes motility and invasion [97]. Finally, it seems that PKCζ plays a very important role in gliomagenesis [97].

#### 4.2.4. JAK/STAT Pathway

Janus kinase (JAK)/signal transducer and activator of transcription (STAT) signaling is known to drive growth, invasion, treatment resistance, stemness maintenance and immunosuppression in gliomas [98]. Among the STAT family, STAT3 is the best characterized in GBM. Its aberrant signaling is mainly the result of dysregulated upstream events and not of gain-of-function mutations [98]. Upstream regulators include not only JAK, but also RTKs such as EGFR, FGFR, and PDGFR [98,99]. Depending on the mutational profile of the tumor, STAT3 could play a dual tumor-suppressive or oncogenic role [100]. As a consequence, inhibition of STAT3 should be considered only in specific cases. More specifically, STAT3 inhibitors may be useful in the treatment of EGFRvIII-expressing GBMs, but not in the treatment of PTEN-deficient tumors [100].

#### 4.2.5. P53 Pathway

The p53 pathway is activated in response to carcinogenesis events such as DNA damage, genotoxicity, and aberrant growth signals, and it is frequently deregulated in GBM [101,102]. The most common mutations are missense mutations in TP53, deletions of CDKN2A/ARF, and/or amplifications of MDM2 and MDM4 [102]. According to the TCGA data, the deregulation of the p53 pathway was found in 86% of GBMs [53]. Loss of p53 results in uncontrolled cell proliferation and tumor progression [103]. However, it is still unclear whether the mutated p53 results in the same oncogenic functions as loss of p53 [102,103].

#### 4.2.6. Rb Pathway

The Rb pathway is involved in the regulation of the cell cycle and, similarly to the p53 pathway, its deregulation leads to tumor progression [104]. Overall, genetic alterations were found in approximately 80% of GBMs [53]. The most frequently altered genes are CDKN2A and CDKN2B, deletions or mutations whereof were detected in 61% of the GBMs. Amplification or mutations in CDK4 or CDK6 could also be detected in approximately 14% and 2% of GBMs, respectively [53]. However, RB alterations were accounted only for 7.6% of the tumors [53]. In fact, it appears that the Rb pathway is preferentially altered at components that lead to RB inactivation by hyperphosphorylation, which leads to suppression of its cell cycle blocker function and sustainment of proliferative signaling [105]. As a matter of fact, CDK inhibitors have being investigated as a novel treatment option for GBM patients [106].

## 5. Small Kinase Inhibitors: Mechanisms of Resistance

Protein kinases have been pursued as potential drug targets for the treatment of cancer, and most of the approved kinase drugs are active against more than one type of tumor [107]. Many of the currently known kinase inhibitors target the ATP binding site with the kinase activation loop in the active (type 1) or inactive (type 2) conformation [108]. Type 3 inhibitors are non-ATP site (allosteric) kinase inhibitors that show the highest degree of selectivity by exploiting binding sites and regulatory mechanisms that are unique to a particular kinase [109]. Several protein kinases are highly disrupted, thus representing attractive therapeutic targets in GBM [110] (Table 1). However, in clinical trials, they have demonstrated very limited efficacy in unselected GBM populations [54].

One of the reasons behind the failure of the therapeutic intervention using small kinase inhibitors may reside in resistance. Not many studies have been conducted on GBM to investigate this hypothesis. Nevertheless, in the next paragraphs, we tried to summarize the major findings supporting the role of resistance in the inefficacy of these treatments.

**Figure 2:**
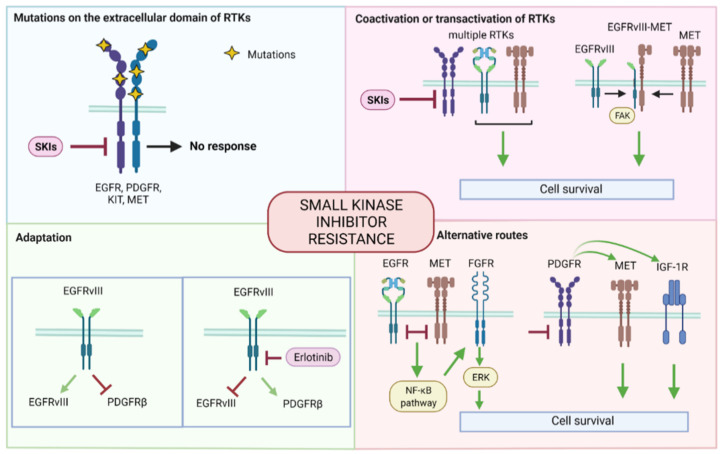
Summary of small kinase inhibitor resistance mechanisms. The mechanisms can be grouped as follows: mutations on the extracellular domain of RTKs, coactivation and transactivation of RTKs, adaption, and alternative routes. SKI: small kinase inhibitor, EGFR: epidermal growth factor receptor, PDGFR: platelet-derived growth factor receptor, c-KIT: stem cell factor receptor, c-MET: mesenchymal epithelial transition factor, RTK: tyrosine kinase receptor, PTEN: phosphatase and tensin homolog, FGFR: fibroblast growth factor receptor, IGF-1R: insulin-like growth factor 1 receptor, NF-κB: nuclear factor kappa-light-chain-enhancer of activated B cells, ERK: extracellular signal-regulated kinase.

Small kinase inhibitors target very specifically one or multiple kinases. As such, the mechanisms in place that overcome their effect are more limited compared to the broad range of TMZ resistance mechanisms. With targeted therapies, four main categories of escape can be identified: the presence of specific mutations, coactivation, adaptation, and activation of alternative routes (Figure 2).

### 5.1. Mutations

Specific mutations in the drug target and other signaling genes are among the main causes of primary resistance. What can be observed when comparing gliomas to other types of tumors, like lung and gastrointestinal (GI) cancers, in terms of efficacy is the presence of a different distribution of mutations in specific gene regions. An example is given by EGFR and its inhibitors. EGFR kinase inhibitors are already FDA-approved drugs for the treatment of lung cancers, but results in GBM have been disappointing [54,120]. Looking at the mutational profile of these two types of tumors, it is worth noting that the EGFR gene in lung cancer harbors alterations at the kinase domain level, while in GBM, they are found predominantly on the extracellular domain [121,122]. Similarly, PDGFR and KIT, the inhibitors whereof are more effective in gastrointestinal stromal tumors (GIST), are frequently characterized by activating mutations in intracellular domains [123,124]. In GBM, however, point mutations have been found in the extracellular region, resulting in an outcome that has not been beneficial [16]. On the other hand, the FGFR–TACC fusion gene has displayed high sensitivity to FGFR inhibitors, and clinical trials are ongoing in recurrent GBMs harboring this mutation [125]. However, even though the necessity of a more personalized therapy and stratification of patients has been widely discussed and assessed, the identification of markers of response still needs to be optimized. Based on the current knowledge on GBM and other types of tumors, it appears that there may be a relationship between the poor efficacy of small kinase inhibitors and mutations restrained on the extracellular domain which may partially explain the primary resistance encountered in GBM.

### 5.2. Coactivation or Transactivation of Other RTKs

Intrinsic resistance can also derive from coactivation of other cellular signals. Concomitant activation of RTKs within individual tumors is commonly found in GBMs, and it has been hypothesized as a crucial mechanism for the maintenance of the tumor, thus rendering GBM refractory to single-agent inhibition [126]. The presence of three or more activated RTKs has been described in individual tumors, including EGFR, ERBB3, PDGFRα, and MET [127]. When labeling multiple RTKs, Szerlip et al. observed that in most cases, distinct tumor cell subpopulations were amplified for only one RTK, while only a small part was amplified for more than one [128]. These findings are in line with the characteristic intratumoral heterogeneity and support the hypothesis of its contribution to the poor response to kinase inhibitor monotherapies. An analysis of the TCGA GBM dataset has revealed a tight relationship of PDGFR with three other RTKs (ERBB3, IGF1R, TGFBR2), the copresence whereof not only enhanced imatinib tolerance, but also promoted migration and invasion [129]. Other results derived from the analysis of EGFR/EGFRvIII and PTEN in recurrent malignant gliomas from patients who received EGFR kinase inhibitors revealed that co-expression of EGFRvIII and PTEN was associated with responsiveness [130]. It was subsequently discovered that the loss of PTEN triggered the opposite effect, promoting resistance to EGFR kinase inhibitors by dissociating EGFR signaling from downstream inhibition [131]. However, PTEN is not the only player linked to EGFR; c-Met could be transactivated by EGFRvIII, involving formation of an EGFRvIII–MET heterodimer with the support of FAK, playing a key role in supporting kinase inhibitor resistance [132]. Moreover, c-Met could induce EGFR activation, creating an important autocrine signal loop that promoted tumor growth and resistance [133,134,135,136]. Therefore, it is not surprising that MET gene amplification is also involved in the EGFR kinase inhibitor resistance in non-small-cell lung cancer (NSCLC) and colon cancer [137,138].

### 5.3. Adaptation

Most tumors, despite initial efficacy, experience the development of drug resistance. In fact, tumors acquire the ability to adapt and escape from the damaging effects of treatments. This type of resistance is remarkably diverse and complex and, for kinase inhibitors in GBM, has not been fully understood or characterized yet. Among the most studied kinases in GBM, EGFR is frequently mutated, giving rise to constitutively active variant EGFRvIII. Nathanson et al. observed an interesting adaptive phenomenon occurring in EGFRvIII-positive GBM cells. By creating erlotinib-resistant GBM cell lines through continuous treatment with the compound, they found that GBM cells suppress EGFRvIII protein expression. Moreover, withdrawal of the drug restored extrachromosomal EGFRvIII DNA and resensitized the tumor cells [139]. EGFRvIII appears to be a plastic player in the adaption to drugs. A persistent active EGFRvIII has been shown to suppress PDGFRβ expression through mTORC1 and ERK signaling. However, its inhibition by erlotinib reactivated PDGFRβ transcription, thus sustaining tumor survival [140]. Additional mechanisms, such as secondary mutations, additional amplifications, extracellular sequestration of drugs, and drug efflux have been proposed mainly for other cancers, but most of them still need to be investigated in GBM [141].

### 5.4. Alternative Routes (Bypass)

Activation of alternative routes subsequent to the administration of a drug is also commonly observed. The acquisition of a bypass signal has been investigated for GBM for both mono- and combinational therapies. As an initial response to combined EGFR and MET inhibition, the NF-κB pathway has been activated. Its activation promoted an autocrine activation of FGFR and reactivation of ERK, driving SPRY2 expression and cellular survival response [142]. Induced resistance with a PDGFR inhibitor on proneural GBM tumors was shown to induce distinct types of RTK bypass activation. Pastorino et al. presented a study on the enhancement of c-Met activation in nilotinib-induced resistant tumors, while a study conducted by Bonnin et al. demonstrated the association of developed resistance to PDGFR inhibition with IR/IGF-1R activation [143,144]. IGF-1R seems to play a significant role in the development of escape routes to targeted therapies in GBM. In fact, it has also been described as a protector against apoptosis and a mediator for resistance to EGFR inhibitors through constant activation of PI3K and AKT signaling [81,145,146]. However, tackling MET or IGF-1R also leads to obstacles. In fact, MET inhibitors have not been effective due to the activation of bypassing signals that included the involvement of mTOR, FGFR1, EGFR, STAT3, and COX-2 [147]. Instead, IGF-1R inhibitors have shown limited efficacy as they give an appropriate response only in combination with other inhibitors, but not as a monotherapy [148,149,150].

### 5.5. Glioblastoma Stem Cells (GSC)

A special mention in chemoresistance should be attributed to a subpopulation of GBM cells characterized by stem cell-like properties with the ability to self-renew and differentiate, constituting the diverse hierarchy of cells composing the tumor [151]. Glioblastoma stem cells (GSCs) are considered to be at the top of the hierarchy of cellular differentiation, characterized by the highest entropy and capacity for adaptation. In this context, it has been hypothesized that therapy resistance of the cancer stem cell population derives from their plasticity, both intrinsically from variations in gene expression and extrinsically from interactions with a variety of external factors such as the TME [152,153]. One of the states characterizing GSCs is the entrance into quiescence. The quiescent state protects these cells, particularly from antiproliferative agents, and is thus an important factor of therapy resistance [154,155]. The quiescent aspect has been investigated in chronic myeloid leukemia (CML) where the integrin-linked kinase (ILK) was pointed out as a survivor mediator critical to tyrosine kinase inhibitors and quiescent stem cells [156]. Other mechanisms, including the ones mentioned in the previous paragraphs, epigenetic modifications, increased drug efflux, epithelial-to-mesenchymal transition, lncRNA, and exosome-mediated cell–cell communications, were suggested to be characteristic of the tumor stem cell-like population in lung and thyroid cancers resistant to kinase inhibitors [157,158]. Therefore, similar aspects could be employed by GSC to circumvent the toxic effects of small kinase inhibitors.

## 6. Overcoming Drug Resistance

### 6.1. In Vitro Models for Drug Resistance

A critical factor that affects the progress in GBM treatment is the establishment of clinically relevant in vitro models to study drug resistance. A better understanding of the processes that drive GBM progression under the current treatment would enable the development of more efficient tackling strategies. On the one hand, in vitro systems allow rapid screening of cells for drug sensitivity and resistance, which could be translated into adjustments to the therapy. On the other hand, they may also provide a source of potential biomarkers that could be used to monitor the evolution of resistance. The crucial variables to consider when developing drug resistance models in vitro are the architecture, the heterogeneity, the microenvironment, and the drug delivery to the tumor cells.

Regarding the architecture, standard 2D cell cultures have been the most used in vitro model. In the past twenty years, the amount of 3D cell culture models used in scientific research has increased exponentially [159]. Nowadays, it is widely accepted that cell responses in 3D cultures are more representative of the in vivo conditions [160]. In fact, the study of cells in a 3D context can provide insights not observed in traditional 2D monolayers, such as cell–cell interaction and exchange of nutrients and metabolites between the core and the periphery of the tumor. Several reviews have already investigated the differences in 2D versus 3D characteristics in terms of morphology, proliferation, gene expression, adhesion, differentiation, apoptosis, and response to stimuli [159,160,161,162,163,164]. In relation to drug response, 3D cultures are characterized by higher resistance and provide a more accurate representation of drug effects than monolayers. In a recent study, response to the treatment of Stupp-treated 2D and 3D GBM cultures showed a higher general resistance in the 3D organoids than in the cells in monolayer [165]. In addition, resistance to multikinase inhibitors was also mediated differently in 3D GBM cell cultures. In particular, the MEK–ERK and PI3K–Akt pathways, but not PDGFR, mediated the dimensionality-dependent chemoresistance [166].

As for the second aspect, as GBM is a highly heterogeneous tumor, it is important to recapitulate its specific characteristics. The use of patient-derived cancer cell cultures brings about a superior advantage in preclinical models over immortalized cancer cell lines. For decades, immortalized cancer cell lines have been the mainstay of cancer research as they offer advantages such as being cost-effective, easy to use, and provide a consistent sample with reproducible results [167]. However, their clinical value as a representative model is being questioned with growing concern as they may not adequately represent primary cells [167,168]. The use of low-passage, serum-free, and patient-derived GBM cell cultures has now been widely accepted as the gold standard for in vitro models as they recapitulate specific genetic features and tumor heterogeneity [169]. In fact, the use of patient-derived cell cultures allows the integration of genomic data with drug sensitivity data, which may lead to identification of predictive signatures and enable future stratification of patients to more effective therapy regimens [170]. In particular, patient-derived orthotopic xenograft (PDOX) models have been proposed as a model for testing therapeutics aimed at preventing GBM recurrence as they allow the recreation of the genetic, histologic, and morphologic profiles of human GBM [171,172]. However, they are still limited by the lack of human stromal and immunological components, which may be partially solved in the future with the use of “humanized” mice [171].

Another important factor in in vitro cancer modeling is the tumor microenvironment, particularly the tumoral niche and the extracellular matrix (ECM) [173]. The components that form the TME in GBM are comprised of different types of cells, such as normal astrocytes and tumor-associated macrophages (TAMs). The incorporation of nonneoplastic astrocytes into coculture systems has already provided insights into their supportive role in GBM resistance [174,175]. Coculture systems with GBM cells and macrophages also demonstrated their crucial role in the modulation of the immunosuppressive environment in the presence of TMZ. In fact, it was observed that chemoresistant glioma cells were more effective than TMZ-sensitive cells in inducing a strong immunosuppressive macrophage M2 polarization, mainly characterized by high IL-10 release, CD206 expression, and arginase activity [176]. ECM composition also plays a major role in GBM behavior. As a matter of fact, GBMs have been found to synthesize and utilize many ECM components, such as tenascin, vitronectin, laminin, fibronectin, and collagen type IV [177]. Tenascin-C is indispensable for the growth of GSCs as well as GBM invasion, which are phenotypes largely contributing to chemotherapy resistance [178]. In a recent study, a bioengineered scaffold for 3D cultures was created to investigate the role of ECM components in promoting chemoresistance in GBM. The cooperative engagement of CD44 through hyaluronic acid (HA) and integrin αV facilitated resistance to alkylating agents by inhibiting the expression of proapoptotic factors [179]. Similarly, GBM cultures on chitosan–hyaluronic acid (CHA) scaffolds enhanced stem cell marker expression and drug resistance [180].

While the previous two variables could be applied for several cell culture model applications, the selection of drug doses and duration of exposure are more specific for the investigation of drug resistance. In fact, the use of high drug concentrations on cell cultures more likely selects for clones that are intrinsically resistant to the therapeutic agent. This approach is useful when investigating the primary type of resistance. However, it excludes the population of cells that acquire resistance during the treatment. A common approach to model-acquired resistance has been by exposing sensitive cancer cells to gradually increasing concentrations of the drug over an extended period of time [181]. Regarding the dose and time of exposure, the method of choice depends on the specific scientific question. However, if working with patient-derived material, it may be important to consider the specific drug sensitivity of each sample in order to use a consistent approach personalized to the patient material. In a recent study on breast cancer, they investigated the effect of different treatment scheduling on drug resistance by tracking clonal dynamics using DNA-integrated barcodes and single-cell RNA sequencing. What their results suggested is that longer formats of treatment schedules for in vitro screening assays are required to understand the effects of resistance [182].

Overall, all these variables need to be taken into account when developing in vitro drug resistance models. In the scientific literature, there is great variability within the identified resistance mechanisms in GBM. Part of this variability could derive from the application of numerous different models and strategies, raising the issue of using more standardized approaches to investigate a multifactorial phenomenon like drug resistance.

### 6.2. Clinical Strategies

**Figure 3:**
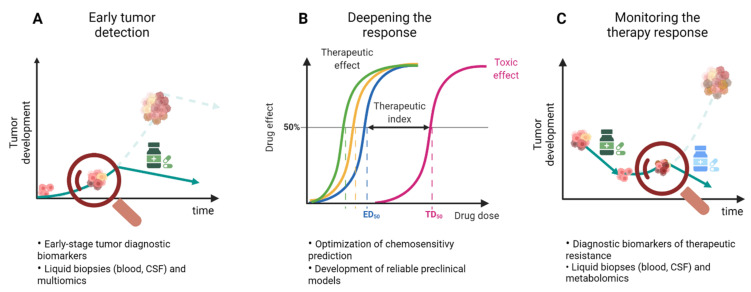
Three strategies to approach drug resistance in GBM. (**A**) Detection of the tumor at early stages would allow a more effective eradication of the tumor and prevention of drug resistance. (**B**) Deepening the drug response by optimizing the chemosensitivity prediction would bring about a more effective and safe therapeutic effect. (**C**) Constant monitoring of the therapy would allow the early detection of resistance which can subsequently be tackled by a second therapy intervention.

In the clinical setting, drug resistance could potentially be tackled in different ways. Vasan et al. proposed various approaches which include (1) earlier detection and cancer interception, (2) deepening therapeutic response, and (3) therapeutic monitoring with adaptive interventions (Figure 3) [183].

In the first case, intercepting cancers at an early stage when the clonal diversity is still low would be more effective in tumor eradication and prevention of resistance (Figure 3A). However, in order to apply this approach, the availability of diagnostic biomarkers and the implementation of screening are essential. In GBM, the detection of biomarkers is of great interest and an advancing research field. In particular, the use of liquid biopsies is appealing as it would provide a minimally invasive method which could be frequently repeated. Liquid biopsies of tumor-specific components include circulating tumor cells (CTCs), circulating tumor DNA (ctDNA) and RNA (ctRNA), circulating microRNAs, proteins, tumor-educated platelets (TEPs), and extracellular vesicles (EVs) [184]. Recently, metabolomics has also been of increased interest and proposes interesting new leads for the development of diagnostic tools. Serum biomarkers reported in the literature are related to vascular proliferation, cell growth, inflammation, immune system, coagulation, and nutrition [185]. The panel of potential diagnostic biomarkers in GBM is rapidly expanding for blood and in particular for cerebrospinal fluid (CSF), as reviewed by Verheul et al. [186]. However, knowledge about the early stages of GBM is still limited and there are no standard diagnostic or therapeutic strategies for this stage at present [184,187]. Currently, screening for GBM has no clinical relevance due to low incidence, lack of sensitive biomarkers, and the apparently de novo development of these tumors within a few weeks or months [185].

The second approach aims to prevent the development of drug resistance by deepening the response to the therapy (Figure 3B). This approach requires the optimization of chemosensitivity prediction to select the most effective treatment. The knowledge of the genomic background becomes essential to identify potential therapeutic targets as well as the implementation of drug screening on the primary material. The utilization of improved and standardized patient-derived cell culture models could provide more physiologically relevant data. As reported by Lippert et al., it has been certified that pretreatment testing of drugs on short-term cultures is useful in the rapid recognition of sensitivity and resistance as it allows treatment decisions or adjustments shortly after [188,189,190]. As previously discussed, the use of an appropriate in vitro model could bring about a great advantage in the understanding of drug resistance mechanisms.

The third approach implies constant monitoring of response to the therapy in order to quickly detect markers of resistance and adjust the current treatment to a more effective second-line intervention (Figure 3C). Identification of molecules that play a role in drug resistance mechanisms is highly attractive as these components could be used as potential biomarkers for the prediction of drug response and to monitor tumor progression. An early diagnosis of drug resistance would in fact improve treatment by enabling earlier modifications in dose, schedules, and therapeutic regimens. Furthermore, in this case, identification of the therapeutic response through liquid biopsies would be ideal. A panel of identified molecules in liquid biopsies of GBM patients in relation to therapy response has been widely documented by Saenz-Antoñanzas et al. They reported MGMT, miR-128, miR-342, miR-205, GAS5, PD-L1, TGM2, CD44, and CD133 as therapy response biomarkers identified in blood samples [184]. In addition, a group of microRNAs consisting of miR125b, miR-223, miR-451, miR-711, miR-935, miR-21, miR-218, miR-193b, miR-331, miR374a, miR548c, miR520f, miR27b, miR-30b miR-10b, and miR-15b was listed in samples derived from cerebrospinal fluid (CSF) [184]. However, these markers are not frequently detected. In a comprehensive study analyzing a cohort of 222 GBM patients, ctDNA alterations were detected only in 55% of the cases [191]. Indeed, identification of biomarkers to monitor tumor evolution and therapeutic response has an enormous potential to improve the clinical management of GBM. However, validation of these discoveries is still necessary.

Regardless of the abovementioned scenarios, the necessity to develop more effective therapies for GBM is of greater urgency. Innovative therapies are being investigated and include many refinements as well as new approaches such as novel tumor growth inhibitors, drug repurposing, tumor-treating fields (TTF), immunotherapy, cell and gene therapy [192]. As there are great interindividual differences in response to treatment, a personalized approach to the management of GBM may be the way forward. With the advent of precision medicine, personalized therapies in clinical practice would in fact suggest a more beneficial outcome for patients. However, a multimodal therapy would probably also be required as no single-agent treatment has thus far been adequate. This raises questions of what the most appropriate therapeutic regimens are and selecting between combinational or sequential application in order to counteract drug resistance. Due to the molecular heterogeneity and redundancy that characterize GBM, the combination of drugs targeting multiple pathways is frequently sought. In preclinical models, many drug combinations are effective and act synergistically for therapeutic benefits; however, the utility of this approach has generally been limited due to overlapping toxicity profiles [193]. On the other hand, a sequential therapy approach may provide for greater dose intensity and treatment time and potentially does not allow treatment-specific side effects to build up. As such, this approach could be more appropriate for frail patients who may not be able to tolerate the toxicity of combinatorial therapy. Furthermore, a second genomic analysis or drug screening of the recurrent tumor may provide relevant information to adjust the therapy with a more appropriate treatment, achieving more effective results. Overall, there is an urgency to investigate further novel combinational and sequential treatments with the goal of achieving a greater therapeutic response while minimizing the toxicity and the occurrence of resistance.

## 7. Conclusions

A considerable effort has been dedicated to understanding the mechanisms of drug resistance in GBM. The well-known intratumoral heterogeneity of GBM is also reflected in the intrinsic and acquired display of therapy resistance. This is particularly true for the standard-of-care chemotherapy compound temozolomide as resistance is mediated by a wide variety of molecular processes which do not comprise only the involvement of tumor cells, but also include a complex network of interactions with its environment as well. Nowadays, cancer treatments are pursuing a more targeted and personalized approach. Nevertheless, these approaches have also shown lacking efficacy in GBM. This is particularly the case of small kinase inhibitors. Their failure in clinical trials has demonstrated once more the strong variety, redundancy, and plasticity of the pathways characterizing this tumor. Due to its multifaceted characteristics, it is comprehensible that a single targeted approach cannot be highly effective.

A more complete understanding of the resistance mechanisms is still necessary to obtain relevant information that could be translated to the clinic. Therefore, it is imperative to push forward with the research aimed at characterizing and overcoming drug resistance. The first step would be the optimization and implementation of improved preclinical resistance models that could provide the first insight on resistance biomarkers. In fact, the detection of resistance at the initial stages is of utmost importance as it could lead to fast therapy adjustments and improved benefits for the patients. In parallel, more sensitive and robust biomarker detection techniques should also be developed and validated in the clinic. Finally, further investigations should also be conducted on alternative personalized therapeutic approaches, such as sequential treatment, as new means to avoid or control the development of drug resistance.

## Figures and Tables

**Table 1:** Summary of the kinases and their small inhibitors evaluated in clinical trials for GBM.

Target Kinase	Small Kinase Inhibitors	Reference
EGFR	Gefitinib, erlotinib, lapatinib, afatinib, dacomitinib, neratinib	[111]
PDGFR	Imatinib, tandutinib, lenvatinib, nintedanib, thyrophostin	[112]
MET	Crizotinib, volitinib, cabozantinib, altiratinib, SGX523, INCB28060, PLB-1001	[69]
FGFR	Dovitinib, nintedanib, lenvatinib, brivanib, orantinib, ponatinib, E3810, ENMD-2076, AZD4547, BGJ398, LY2874455	[73]
VEGFR	Imatinib, cediranib, pazopanib, sorafenib, sunitinib, vandetanib, vatalanib, AEE788, CT-322, XL184	[113,114]
BRAF	Sorafenib, vemurafenib, dabrafenib, encorafenib	[115,116]
MEK	Combimetinib, trametinib, binimetinib	[116]
PI3K	Pictilisib, buparlisib, pilaralisib, sonolisib, dactolisib, voxtalisib, PQR309	[117]
AKT	Perofisine, MK2206	[118,119]
mTOR	Sirolimus, everolimus, temsirolimus, ridaforolimus, onatasertib, dactolisib, voxtalisib, PQR309, gedatolisib, sapanisertib	[117,118]
PKCβ	Enzastaurin	[97]
